# Sequencing of messenger RNA in the healing process of diabetes foot ulcer

**DOI:** 10.3389/fendo.2024.1468301

**Published:** 2024-11-19

**Authors:** Guili Wang, Ding Wu, Donglin Lu, Huifang Wu, Yunmin Cai, Qingyi Meng, Zhaoxuan Liu

**Affiliations:** ^1^ Department of Vascular Surgery, Affiliated Jinan Central Hospital of Shandong First Medical University, Jinan, China; ^2^ Department of Vascular and Wound Center, Jinshan Hospital, Fudan University, Shanghai, China

**Keywords:** diabetic foot ulcers, wound healing, messenger RNA, diabetes, diabetic foot ulcer (DFU)

## Abstract

**Background:**

Transcriptome analysis of skin wound tissues from diabetic foot ulcer (DFU) patients to assess changes in the microenvironment during wound healing is performed by messenger RNA (mRNA) sequencing.

**Methods:**

All 5 patients with initial DFU area ≥ 3 cm^2^ were selected for wound specimen collection at two time points of 0% and 50% wound healing. A total of 10 skin wound samples were obtained for mRNA sequencing. According to the sequencing results, quantitative polymerase chain reaction (qPCR) validation was performed on 12 relevant genes related to angiogenesis, fibroblast proliferation, and wound inflammation. All patients received electrospun poly (L-lactide-co-caprolactone) and formulated porcine fibrinogen (PLCL/Fg) dressing for DFU treatment.

**Results:**

The mRNA sequencing results of DFU skin specimens showed that compared to the 0% and 50% wound healing time points, there were 4347 differentially expressed genes, including 2827 upregulated genes and 1520 downregulated genes. Enrichment analysis of the differentially expressed genes using Gene Ontology (GO) and Kyoto Encyclopedia of Genes and Genomes (KEGG) revealed that the upregulated genes were mainly associated with biological processes such as cell adhesion, adhesion junctions, epidermal development, and skin barrier formation. The qPCR analysis results indicated that the increased expression of fibroblast growth factor, vascular endothelial growth factor, and CD200 gene was related to DFU healing.

**Conclusion:**

The healing process of DFU wounds involves the interaction of multiple factors, especially in inflammation control, angiogenesis, and fibroblast proliferation.

## 1 Introduction

In 2020, approximately 9.3% of adults globally had diabetes; and it is estimated that by 2045, this number will rise to nearly 11% (1). Diabetes is one of the most common chronic diseases in the world today, with high mortality rates and complex complications. Diabetic foot ulcer (DFU) is a severe complication of diabetes and one of the most common complications in diabetic foot patients. Around 15% of diabetes patients will develop DFU, with 84% of these patients undergoing some degree of amputation. In addition to facing significant physical and mental challenges, diabetes patients also bear the financial burden of DFU treatment (2, 3). While clinical methods such as offloading, debridement, and infection control can help treat DFU, the disease remains prevalent and poses risks of amputation or death for patients (4). The pathophysiological relationship between diabetes and impaired skin wound healing is complex. Cells that play a key role in wound healing, when their activity is diminished, can lead to impaired tissue repair in diabetic wounds (5). Keratinocytes and fibroblasts isolated from diabetic foot ulcer wounds exhibit lower proliferative potential and weaker growth factor production capabilities (6). Wound healing is a complex natural process that involves the coordinated actions of various types of cells, enzymes, cytokines, proteins, and hormones. It involves a series of cellular interactions and biochemical processes to repair damaged skin wounds in the human body (7). The process of skin wound repair includes hemostasis, inflammation, cell migration/cell proliferation, and remodeling. The orderly progression of the healing process facilitates the rapid closure of wounds, resulting in well-formed skin morphology or acceptable scarring in the case of acute wounds (8). Chronic wound healing is relatively slow, which is common among diabetic patients. Factors such as persistent inflammation and peripheral nerve damage can exacerbate non-healing wound conditions, increasing the risk of complications. The mechanisms underlying delayed wound healing are multifactorial, including prolonged inflammatory phases and delayed proliferation and remodeling phases. Research by Declue et al. indicates that the delay in diabetic wound healing is associated with the excessive release of pro-inflammatory cytokines such as IL-1β, IL-6, and TNF-α (9). The study by Qiu et al. revealed that diabetic patients with high blood sugar levels exhibit impaired cell proliferation during the wound healing process, along with reduced production of collagen and growth factors (10). Reduced angiogenesis, accompanying decreased levels of growth factors like VEGF and TGF-β1, is also an important reason for impaired wound healing in diabetic patients (11). Rapid and effective wound healing can reduce the adverse complications of DFU. Considering the significant impact of DFU on medical costs and patient survival, there is an urgent need to find new or more effective treatment methods (12). All 5 patients received electrospun poly (L-lactide-co-caprolactone) and formulated porcine fibrinogen (PLCL/Fg) dressing treatment for DFU. Our research group aims to collect skin wound tissues from DFU patients, conduct mRNA sequencing, and perform qPCR analysis to explore the microenvironmental changes and mechanisms of DFU healing.

## 2 Materials and methods

### 2.1 Research protocol

The study was conducted at the Vascular & Wound Clinic of Jinshan Hospital, Fudan University, Shanghai, China, from July 1, 2023, to December 26, 2023. All enrolled patients had type 2 diabetes mellitus with concomitant diabetic foot ulcers (DFU). Ethical approval was obtained from the ethics committee of Jinshan Hospital affiliated with Fudan University (authorization no. JIEC 2021-S44). The experiment followed the principles of the Helsinki Declaration (13), and all participants were required to sign a written informed consent form upon enrollment.

### 2.2 Materials preparation

We collected 5 DFU patients’ wound specimens that met the inclusion criteria: 1. Initial wound area ≥ 3 cm^2^, 2. Age between 30-80 years old, 3. Duration of DFU wound > 1 month, 4. Stable blood sugar control during hospitalization, 5. Ankle-brachial index (ABI) of the affected limb with DFU > 0.6. Exclusion criteria included patients with wound infections, osteomyelitis, or other diseases that could affect wound healing, such as deep vein thrombosis, systemic lupus erythematosus, or any other systemic inflammatory diseases. Patients with severe limb ischemia, defined as unable to palpate the dorsalis pedis artery or ABI < 0.6, were also excluded. Standard wound care that ulcer wound cleaning and dressing changes were performed, the all wounds were covered with PLCL/Fg dressing of equal size to the wound. At the beginning of the experiment and during each dressing change follow-up, the principal researcher took photos and recorded the wounds. The trial lasted for 12 weeks after the patient was enrolled. According to the specific condition of the wound, participants received PLCL/Fg dressing 1 to 3 outpatient dressing changes per week. Samples were taken at 0% and 50% wound healing, with tissues from the bottom and edges of each DFU wound included at each sampling time. A total of 10 wound samples were obtained from the 5 patients for mRNA sequencing and qPCR analysis. The measurement of wound size was conducted using ImageJ software.

### 2.3 RNA extraction and library construction

Use TRIzol reagent to extract total RNA according to the manual. Use NanoDrop 2000 spectrophotometer (Thermo Scientific, USA) for RNA purity and quantification, and use Agilent 2100 Bioanalyzer (Agilent Technologies, Santa Clara, CA, USA) to evaluate RNA integrity. Construct a transcriptome library using the VAHTS Universal V5 RNA-seq Library Prep kit according to the product manual. RNA Quality Check: Ensure the RNA meets the quality and quantity criteria for library preparation. mRNA Isolation: Enrich for mRNA using magnetic beads linked to oligo(dT) primers. First-Strand cDNA Synthesis: mRNA is reverse transcribed to cDNA using random hexamer primers. Second-Strand cDNA Synthesis: The cDNA is synthesized into double-stranded DNA. End Repair and A-Tailing: The double-stranded cDNA is repaired and tailed with an ‘A’ nucleotide to facilitate adapter ligation. Adapter Ligation: Dual indexing adapters are ligated to the cDNA fragments. PCR Amplification: The adapters-ligated cDNA is amplified to enrich the library for sequencing. Library Qualification: The library is quantified and its quality is evaluated using qPCR and/or bioanalyzer before sequencing. These detailed steps ensure comprehensive RNA extraction, assessment, and library preparation, facilitating high-quality RNA sequencing data.

### 2.4 mRNA sequencing

The Illumina Novaseq 6000 sequencing platform was used to sequence the libraries, generating 150 base pair paired-end reads. Each sample obtained 52.21 ± 3.27 million raw reads. The raw data in fastq format was processed using the fastp software to obtain clean reads by removing low-quality reads for subsequent data analysis (14). The HISAT2 software was used for reference genome alignment, and gene expression levels (FPKM) were calculated (15). The read count of each gene was obtained using HTSeq-count.112 The gene count data was analyzed using the R software (version 3.2.0) for Principal Component Analysis (PCA) and visualization to evaluate the intra-group similarity and inter-group differences of the samples. DESeq2 software was employed for differential gene expression analysis, where genes meeting the criteria of q < 0.05 and fold change > 2 or fold change < 0.5 were defined as differential expression genes (DEGs) (16). DEGs were subjected to hierarchical cluster analysis using R software to display the gene expression patterns in different sample groups. The top 30 genes were visualized using the ggradar package in R software to show the expression changes of upregulated or downregulated genes in a radar plot. Subsequently, gene ontology (GO) and Kyoto Encyclopedia of Genes and Genomes (KEGG) enrichment analysis of DEGs were performed based on hypergeometric distribution algorithm to identify significantly enriched functional terms (17, 18). Differential gene significant enrichment results were visualized using bar charts, chord diagrams, or enrichment analysis bubble charts in R software.

### 2.5 qPCR experimental

Validation of 12 related genes in vascular neogenesis, fibroblast proliferation, and wound inflammation based on mRNA sequencing differential gene results and previous research on hot genes was performed using qPCR. The specific genes validated were: ACTA2, VEGFC, PDGFA, FGFR2, COL4A2, COL7A1, TNF, IL6, IL10, CD68, CD200, and CD163.

#### 2.5.1 RNA extraction

Total RNA was extracted and quantified using NanoDrop 2000 spectrophotometer (Thermo Scientific, USA) to determine concentration and OD260/OD280 ratio, followed by RNA integrity assessment via agarose gel electrophoresis.

#### 2.5.2 Reverse transcription

The target RNA was reverse transcribed into cDNA using the TransScript All-in-One First-Strand cDNA Synthesis SuperMIX for qPCR kit. The reverse transcription system included: total RNA, 0.5 μg; 5×TransScript All-in-one SuperMix for qPCR, 2 μl; gDNA Remover, 0.5 μl; Nuclease-free H2O up to 10 μl. The reaction conditions were: 42°C for 15 minutes, followed by 85°C for 5 seconds. After reverse transcription, the cDNA was diluted with 90 μl Nuclease-free H2O and stored at -20°C for later use.

#### 2.5.3 Quantitative PCR

The PCR reaction was conducted on a LightCycler^®^ 480 II real-time PCR system (Roche, Swiss) using the PerfectStartTM Green qPCR SuperMix kit (Contains all components required for qPCR reactions, including Taq DNA polymerase, dNTPs, buffer, and fluorescent dye). The reaction components included: 2×PerfectStartTM Green qPCR SuperMix, 5 μl; 10 μM Forward primer, 0.2 μl; 10 μM Reverse primer, 0.2 μl; cDNA, 1 μl; Nuclease-free H2O, 3.6 μl. The PCR program consisted of: 94°C for 30 seconds; followed by 45 cycles of 94°C for 5 seconds, 60°C for 30 seconds. After cycling, a melting curve analysis was performed to verify product specificity by gradually increasing the temperature from 60°C to 97°C with fluorescence signal collection at each degree.

#### 2.5.4 The expression levels of mRNAs were normalized to GAPDH and were calculated using the 2-ΔΔCt method

## 3 Results

### 3.1 DFU treatment

**Figure 1:**
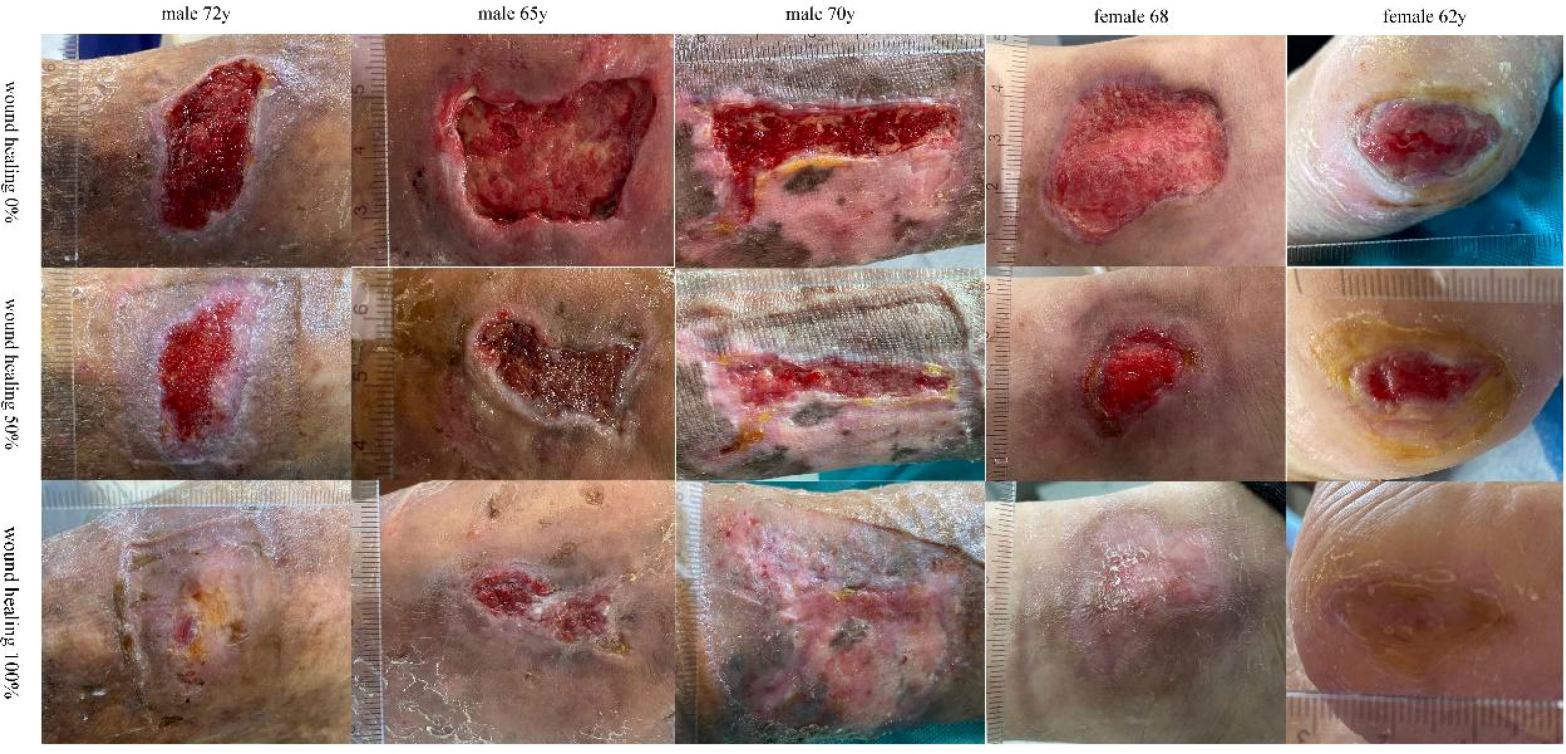
The wound condition of DFU at wound healing 0% 50% and 100%.

The baseline characteristics and DFU features of the 5 enrolled patients are shown in **Table 1**. After 6-12 weeks of treatment, all patients’ DFU wounds healed without any PLCL/Fg dressing-related complications. Two patients achieved complete healing of DFU wounds by the sixth week of treatment (Characteristic 3 and 5). One patient showed substantially healed DFU wounds by the end of the 12-week experimental period, with only local granulation tissue still exposed (Characteristic 2). The **Figure 1** shows that wound conditions of DFU at two sampling time points and the healing status of the wound.

**Table 1:** Demographic and ulcer characteristics of the participant.

Characteristic	1	2	3	4	5
Age, year	72	65	70	68	62
Sex	male	male	male	female	female
Height, cm	165	173	152	158	160
Weight, kg	68	62	50	53	59
BMI	25	20.7	21.6	21.2	23
Duration of diabetes, year	10	8	8	15	6
Walking status	walking	walking	walking	walking	Wheelchair
Target ulcer leg	left	right	left	right	left
Target ulcer location	malleolus	dorsum	malleolus	malleolus	heel
Target ulcer age, week	12	10	9	10	8
Wagner grade	grade I	grade I	grade I	grade I	grade I
DFU extent, cm^2^	5.1	7.4	5.8	8.6	3.4
Healing time, week	7	12	6	10	6

### 3.2 Cluster analysis and screening of differentially expressed genes

**Figure 2:**
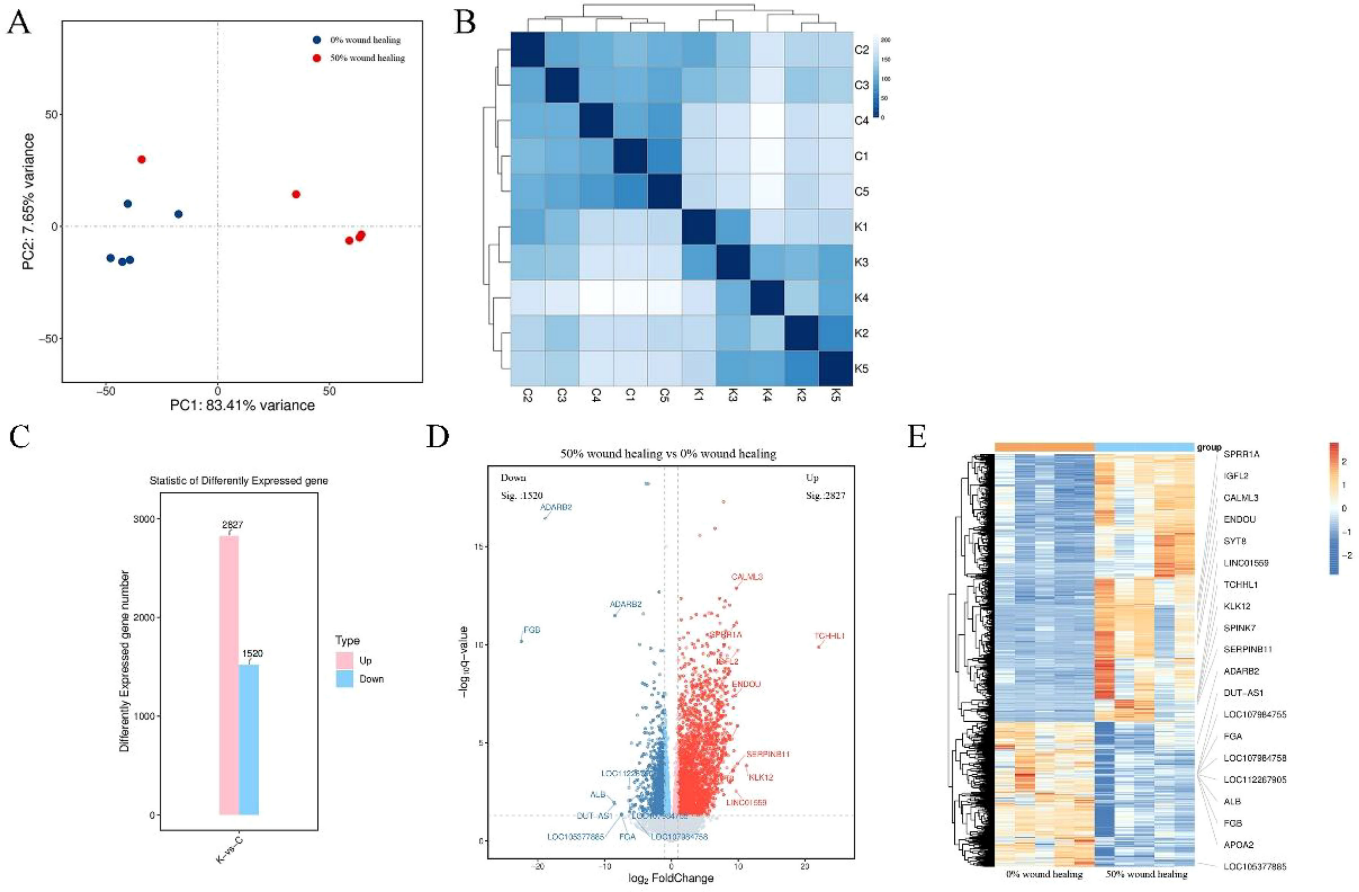
Clustering analysis and differential gene screening of two groups. **(A)** Principal component analysis of 10 samples. **(B)** Cluster analysis of 10 samples. C1-C5 represent wound healing 0%, K1-K5 represent wound healing 50%. **(C)** Overview of differential genes. **(D)** Volcano plot of differential genes, gray represents non-significant differential genes, red and blue represent significant differential genes. **(E)** Heatmap of differential gene clustering. Labeling the top 10 upregulated and top 10 downregulated genes.

For DFU wound samples at two time points, PCA shows significant differences. The samples of 0% wound healing (blue dots) cluster well, while those of 50% wound healing (red dots) are more dispersed but can still be classified effectively (**Figure 2A**). Cluster analysis is used to analyze the differences between samples and study their similarity. The analysis results accurately reflect the origin of the experimental samples, with samples from the same source or time point shown as darker blue and clustered together in **Figure 2B**. The 5 samples from 0% wound healing and 5 samples from 50% wound healing show significant differences between them, while demonstrating good similarity within each group. **Figure 2C** illustrates that there are 4347 DEGs between the samples of 0% wound healing and 50% wound healing, including 2827 upregulated genes and 1520 downregulated genes. The distribution of differentially expressed genes (DEGs) reveals significant differences between the two groups of samples, which are reflected in a volcano plot as shown in **Figure 2D**. The top 10 most significantly upregulated and downregulated genes are separately labeled. **Figure 2E** is a heatmap of differentially expressed gene grouping and clustering based on the expression levels of DEGs. It can reflect the expression patterns of DEGs. The results indicate that there are clear clustering differences in the expression of DEGs between the two groups of samples, confirming both the similarity within groups and the differences between groups.

### 3.3 GO enrichment analysis

**Figure 3:**
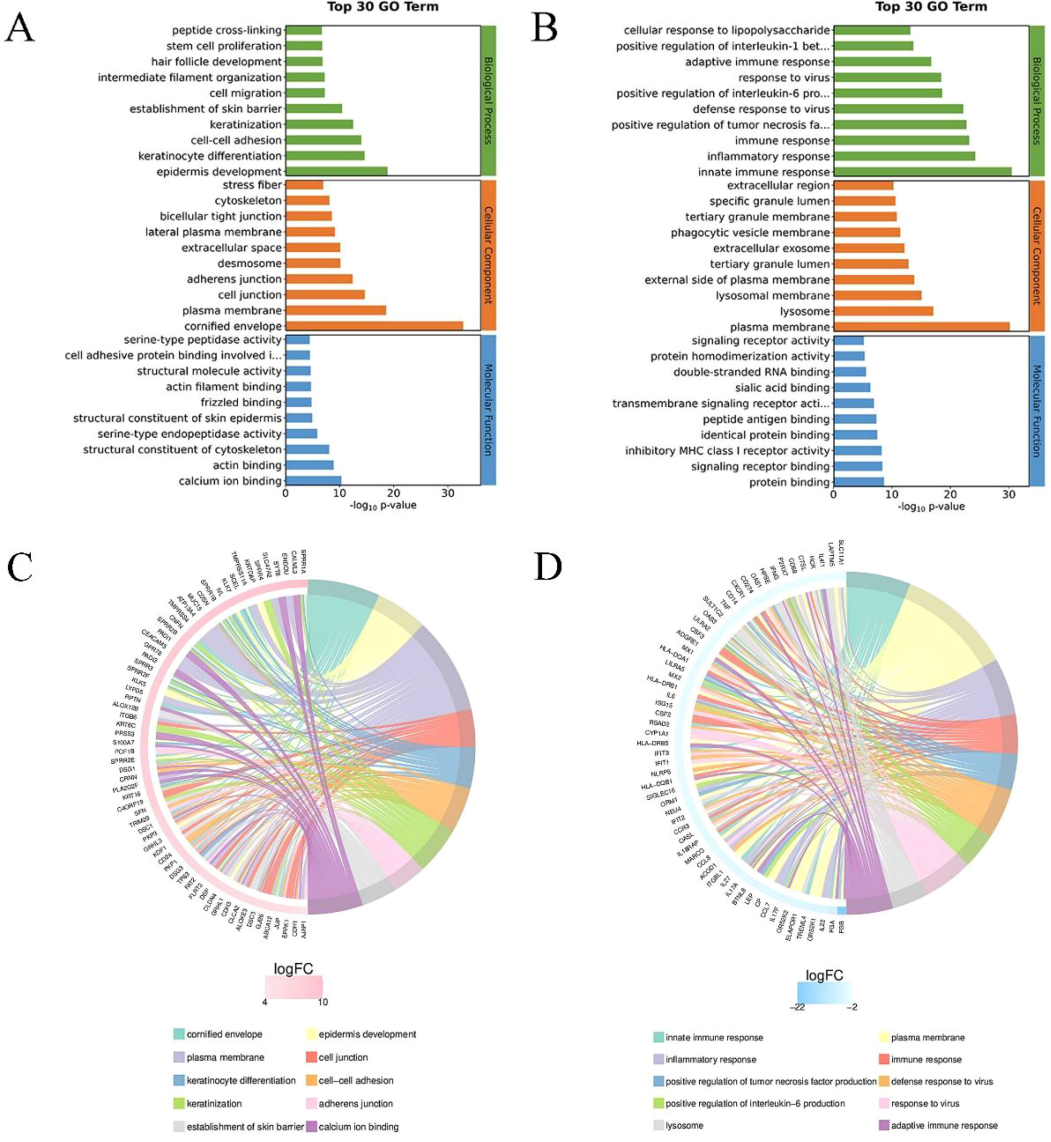
GO enrichment analysis of differential genes. **(A)** Top 30 GO enrichment terms for upregulated differential genes. The y-axis represents the GO term names, and the x-axis represents -log10p-value. **(B)** Top 30 GO enrichment terms for downregulated differential genes. **(C)** Chord diagram analysis of the top 10 GO enrichment terms for upregulated differential genes. The left side shows genes with larger |logFC| in each category, the right side reflects the composition of categories, and the lines in the middle indicate the correspondence between categories and genes. **(D)** Chord diagram analysis of the top 10 GO enrichment terms for downregulated differential genes.

Perform GO enrichment analysis on the obtained differential genes and describe the associated functions. Calculate the results to obtain a significant enrichment p-value (calculate each item in biological process, cellular component, and molecular function using Fisher exact test), the lower the value, the more statistically significant the difference. **Figures 3A, B** respectively list the top 30 items of upregulated and downregulated genes in the GO analysis. The lengths of the bands in three colors represent the importance and difference of the corresponding enriched items. GO enrichment analysis and chord diagram showing the top 10 categories with the smallest q-value or p-value. Similarly, the analysis is based on both upregulated differential genes and downregulated differential genes. The chord diagram in **Figure 3C** displays the upregulated genes and their corresponding GO biological processes, such as cell adhesion, junction organization, epidermis development, and skin barrier establishment. Additionally, the chord diagram in **Figure 3D** shows the downregulated genes and their corresponding GO biological processes, such as inflammatory response, immune system processes, and immune response.

### 3.4 KEGG enrichment analysis

**Figure 4:**
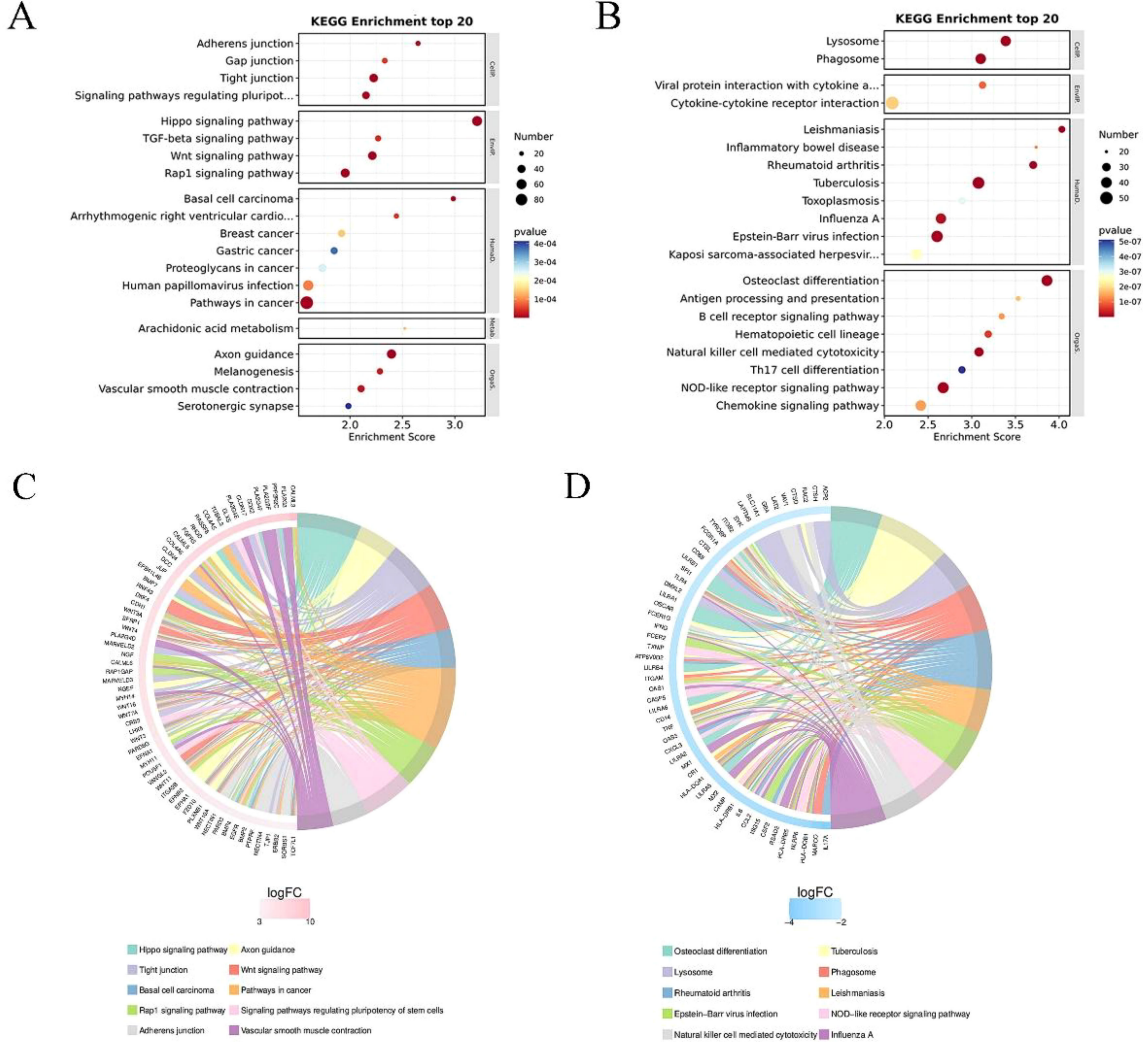
KEGG enrichment analysis of differential genes. **(A)** Results of the top 20 entries for upregulated differential genes. **(B)** Results of the top 20 entries for downregulated differential genes. **(C)** Chord diagram analysis of the top 10 KEGG enrichment terms for upregulated differential genes. The left side shows genes with larger logFC in each category, the right side reflects the composition of categories, and the lines in the middle indicate the correspondence between categories and genes. **(D)** Chord diagram analysis of the top 10 KEGG enrichment terms for downregulated differential genes.

KEGG enrichment analysis top 20 (filtering entries corresponding to PopHits ≥ 5 pathways, sorted in descending order by -log10p-value for each entry), as shown in **Figure 4**. The x-axis represents the enrichment score, with the size of the bubble indicating the number of differential protein coding genes contained in each entry. The bubble color changes from blue-white-yellow-red, with smaller enrichment p-values (red) indicating greater significance for that entry. The chord diagram of KEGG enrichment analysis displays the top 10 classification entries with the smallest q-value or p-value. Similarly, it focuses on analyzing both upregulated and downregulated differential genes. In **Figure 4C**, the chord diagram shows the upregulated genes and their corresponding KEGG entries, such as tight junctions, vascular smooth muscle contraction, Wnt signaling pathway, and pluripotent stem cell regulation pathway. In **Figure 4D**, the chord diagram shows the downregulated genes and their corresponding KEGG entries, such as lysosomes, phagosomes, and cell cytotoxicity mediated by natural killer cells.

### 3.5 qPCR analysis

**Figure 5:**
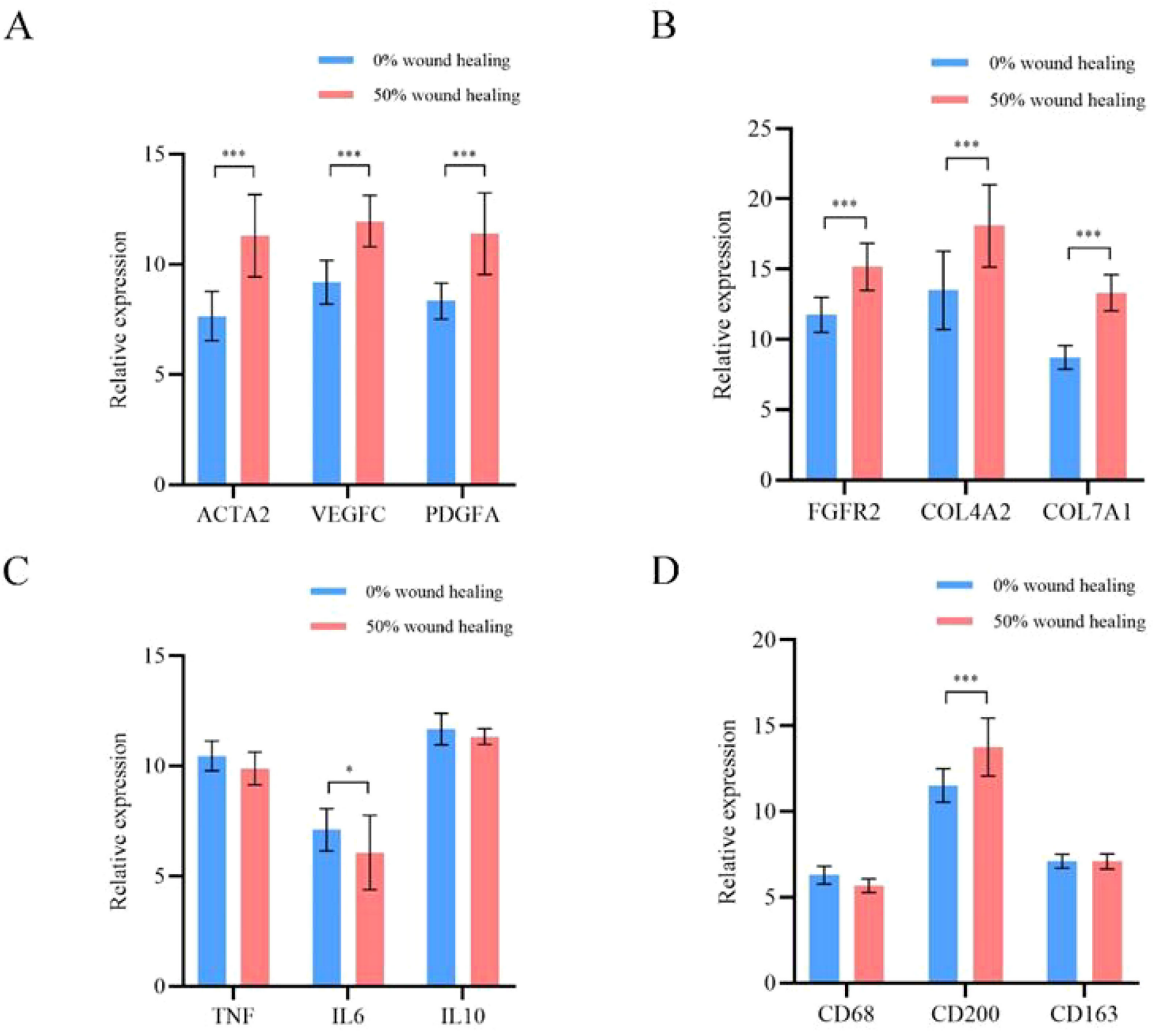
qPCR analysis results of 12 representative differential genes. **(A)** Genes related to angiogenesis. **(B)** Genes related to fibroblast proliferation. **(C)** Genes related to wound inflammation. **(D)** Genes related to macrophage phenotype. *p<0.05, ***p<0.001.

Based on mRNA sequencing results, we selected 12 representative differential genes for qPCR validation. They are: vascular neogenesis ACTA2, VEGFC, and PDGFA; fibroblast proliferation FGFR2, COL4A2, and COL7A1; wound inflammation TNF, IL6, and IL10; macrophage phenotype CD68, CD200, and CD163. Specific quantitative analysis results are shown in **Figure 5**. Among them, there is a statistical difference between the two groups for six representative genes in the directions of vascular neogenesis and fibroblast proliferation, with P-values less than 0.001. CD200 is a marker gene for M2 macrophages, and its expression is higher at the 50% wound healing time point. For the selected inflammatory representative genes, their expression decreases with wound healing, and there is a statistical difference between the two groups for IL6 (P < 0.05).

## 4 Discussion

We sampled wounds from 5 patients treated with PLCL/Fg dressings at two time points, 0% wound healing and 50% wound healing, to study changes in the wound microenvironment through mRNA sequencing and qPCR analysis. Principal component analysis and clustering analysis revealed significant mRNA expression changes between the two groups of wounds, with good similarity between samples within each group. As DFU patients’ wounds healed, there were significant differences in their wound microenvironment compared to the initial state. The study of differentially expressed genes showed significant differences between the two groups of samples, with 4347 statistically significant DEGs, including 2827 upregulated genes and 1520 downregulated genes. The heat map of clustered differentially expressed genes (**Figure 2E**), based on their expression levels, showed distinct clustering differences between the two groups of samples, confirming both intra-group sample similarity and inter-group sample differences.

GO enrichment analysis and KEGG pathway analysis revealed functional differences in differentially expressed genes between 0% and 50% wound healing, involving biological processes, cellular components, and molecular functions (**Figure 3**). GO enrichment analysis and chord diagrams were conducted based on upregulated and downregulated differentially expressed genes. The chord diagram in **Figure 3C** shows upregulated genes and their corresponding GO biological processes, such as cell adhesion, focal adhesion, epidermal development, and establishment of skin barrier. The enhancement of cellular junction manifests as enhanced intercellular communication or structural reinforcement. The upregulation of genes related to epidermal development may indicate the growth or regeneration process of the epidermis, which further affects the establishment of the skin barrier function and is associated with the enhancement of skin protection functions (19). While the chord diagram in **Figure 3D** displays downregulated genes and their corresponding GO biological processes, such as inflammatory response, immune system processes, and immune response. Chord diagrams for KEGG pathway analysis (**Figures 4C, D**) highlight pathway enrichment of upregulated and downregulated differentially expressed genes. Upregulated genes and their corresponding KEGG entries include tight junctions, vascular smooth muscle contraction, Wnt signaling pathway, and pluripotency of stem cells regulation. The enhancement of tight junctions may affect the barrier function between cells, and the increased contraction of vascular smooth muscle may be related to vascular function. The regulation of stem cell function may influence tissue regeneration and repair (20). While downregulated genes and their corresponding KEGG entries, such as lysosomes, phagosomes, and natural killer cell-mediated cytotoxicity. With the healing of DFU wounds, the ability of wound cells to clear foreign substances and metabolic waste decreases (21). It is evident that as DFU wounds heal, pathways such as inflammation and wound clearance are gradually downregulated, while pathways related to wound repair and vascular regeneration are enhanced. These findings are consistent with the study by Rastogi et al., where the use of PLCL/Fg dressings alleviated diabetes-induced wound inflammation by inhibiting inflammatory signaling pathways such as IL-17 and TNF, promoting the transition of macrophages from M1 to M2 phenotype, thereby accelerating wound healing (22).

Previous studies have shown that angiogenesis is an important process in wound healing (23–25). The changes in the wound microenvironment during DFU healing are multifaceted, involving wound nutrition support, clearance of metabolic waste, and skin structure regeneration (26, 27). Faster and better angiogenesis is crucial for skin tissue reconstruction (28). We selected 12 differentially expressed genes from three key research directions for qPCR quantitative validation. They are: vascular neogenesis genes ACTA2, VEGFC, and PDGFA; fibroblast proliferation genes FGFR2, COL4A2, and COL7A1; wound inflammation genes TNF, IL6, and IL10; and macrophage phenotype genes CD68, CD200, and CD163. Specific quantitative analysis results are shown in **Figure 5**. Among them, there is a statistical difference between the six representative genes in the study directions of angiogenesis and fibroblast proliferation, with P<0.001. CD200, a marker gene for M2-type macrophages, shows higher expression at 50% wound healing time point. For the selected inflammatory representative genes, their expressions decrease as the wound heals, and IL6 shows statistical difference between the two groups (P<0.05). It can be observed that during the DFU wound healing process, there is downregulation of inflammation genes and M1 macrophage expression genes, and upregulation of genes related to angiogenesis, fibroblasts, collagen fibers, and M2 macrophages. The results of this study indicate that the angiogenesis-promoting genes α-SMA, VEGFC, and PDGFA are significantly upregulated at the 50% wound healing time point. Additionally, fibroblast proliferation and collagen deposition are crucial for promoting cell proliferation, differentiation, and wound healing (29, 30). Firstly, the changes in the microenvironment of DFU wounds during healing are multifaceted. Secondly, the PLCL/Fg skin regeneration rapidly initiating wound healing from multiple aspects, including angiogenesis, fibroblast proliferation, and alleviation of wound inflammation.

## 5 Conclusion

Through mRNA sequencing analysis, we found that the changes in the wound microenvironment during DFU healing are multifaceted. Pathways such as cell adhesion, tight junctions, epidermal development, and vascular smooth muscle contraction are upregulated, while pathways related to inflammation response and immune response are downregulated. PLCL/Fg dressings can effectively improve the microenvironment of DFU wounds, promoting rapid wound healing.

## Data Availability

The raw data supporting the conclusions of this article will be made available by the authors, without undue reservation.
